# The Application of Australian Rights Protections to the Use of Hepatitis C Notification Data to Engage People ‘Lost to Follow Up’

**DOI:** 10.1093/phe/phae006

**Published:** 2024-05-17

**Authors:** Freya Saich, Shelley Walker, Margaret Hellard, Mark Stoové, Kate Seear

**Affiliations:** Burnet Institute; Burnet Institute; Curtin University; Monash University; Burnet Institute; The Alfred Hospital; Monash University; The University of Melbourne; Burnet Institute; Monash University; La Trobe University; La Trobe University

## Abstract

Hepatitis C is a global public health threat, affecting 56 million people worldwide. The World Health Organization has committed to eliminating hepatitis C by 2030. Although new treatments have revolutionised the treatment and care of people with hepatitis C, treatment uptake has slowed in recent years, drawing attention to the need for innovative approaches to reach elimination targets. One approach involves using existing notifiable disease data to contact people previously diagnosed with hepatitis C. Within these disease surveillance systems, however, competing tensions exist, including protecting individual rights to privacy and autonomy, and broader public health goals. We explore these issues using hepatitis C and Australia’s legislative and regulatory frameworks as a case study. We examine emerging uses of notification data to contact people not yet treated, and describe some of the ethical dilemmas associated with the use and non-use of this data and the protections that exist to preserve individual rights and public health. We reveal weaknesses in rights protections and processes under Australian public health and human rights legislation and argue for consultation with and involvement of affected communities in policy and intervention design before notification data is used to increase hepatitis C treatment coverage.

## Introduction

Hepatitis C is a blood-borne virus affecting 56 million people worldwide (Blach *et al.,* 2022). In many countries, particularly those classified as high income, the virus is transmitted through the sharing of injecting equipment. In 2016, along with 193 member countries of the World Health Organization (WHO), Australia committed to eliminating hepatitis C as a public health threat by 2030 (WHO, 2016). The WHO has set targets to reduce hepatitis C infections for people who inject drugs from 8 per 100 people in 2020 to 2 per 100 people in 2030 (WHO, 2016). Furthermore, a goal has been set to reduce the percentage of people diagnosed with hepatitis C from 30 per cent in 2020 to 90 per cent in 2030, and those treated from 30 per cent in 2020 to 80 per cent in 2030 (WHO, 2016).

Many have argued that achieving elimination targets in Australia is entirely possible (if treatment uptake is increased) given the Australian Government’s decision in 2016 to subsidise new curative hepatitis C treatments, known as direct-acting antivirals (DAAs), which allow decentralised prescribing of treatment through primary care and in prisons (e.g. Dore, 2021; Scott *et al.,* 2020). While it is estimated that approximately 80 per cent of people with hepatitis C in Australia have been diagnosed (Gastroenterological Society of Australia, 2020), declining treatment rates over recent years suggest that many of those previously diagnosed and not yet treated are likely to be ‘lost to follow up’. Those who have been ‘lost to follow up’ are often considered ‘hard to reach’ people that may require more direct and active engagement to encourage them into hepatitis C care (Kracht *et al.,* 2020).

Since 2016, nearly 90,000 people have been treated for hepatitis C in Australia accounting for approximately half of those who were estimated to have hepatitis C at the end of 2015 (Burnet Institute and Kirby Institute, 2021). As Australia moves to the final phases of hepatitis C elimination, the strategies used to treat the first 50 percent are unlikely to be effective for the remaining 50 percent (Pedrana *et al.,* 2021). As a result, attention is being directed to new and innovative approaches to increase the number of people who are tested and treated for hepatitis C. One potential approach involves utilising existing notifiable disease surveillance data to contact people prospectively or previously diagnosed with hepatitis C who are not engaged in hepatitis C care to discuss their diagnosis and offer pathways to care. However, concerns have been raised about the potential for these approaches to limit individuals’ rights in the pursuit of broader public health interests (Buchbinder *et al.,* 2020; Sweeney *et al.,* 2013).

In this article, we briefly describe the historical tensions that have existed within disease surveillance systems between protecting individual rights to privacy and autonomy and broader public health goals. Using hepatitis C and Australia’s legislative and regulatory frameworks as a case study, we explore emerging approaches to using notification data to contact people who have been diagnosed with chronic hepatitis C but not yet received treatment, and some of the ethical dilemmas identified. We then examine these dilemmas in the context of rights protections and processes that exist under Australian public health and human rights legislation to draw further attention to these ethical dilemmas, as well as the limitations of rights protections in Australian notifiable disease legislation. Finally, we pose issues that must be worked through before notification data can be used in this way.

## Context

Since the emergence of compulsory disease notification systems, tensions and controversy have existed between individual rights to privacy and liberty versus public health benefits (Gilbert *et al.,* 2019; Lee *et al.*, 2012). Disease notifications emerged at a time of demographic transition and growing understanding of the causes of disease (Hardy, 1993). For example, in the UK the *Sanitary Act 1866* provided sanitary authorities the power ‘to order the cleaning and disinfection of premises’ (Hardy, 1993: 6). This was a critical point, as ‘the tradition of Englishman’s [sic] liberty and privacy, of his [sic] home as his [sic] castle, was one the legislature was reluctant to breach, especially in respect of the well-to-do’ (Hardy, 1993: 6). Following this, national notifications of infectious diseases became mandatory throughout England and Wales after the passing of the *Infectious Disease* (*Notification*) *Act* 1889 (Ferson, 2013).

The introduction of compulsory disease notifications in the UK (from 1889) and throughout the USA (by 1901) largely aimed to facilitate the quarantine of exposed individuals and the isolation of people with infectious diseases to prevent further spread (Buchan, 1927; Declich and Carter, 1994). However, compulsory disease notifications were met with opposition from the medical profession. Clinicians in the UK claimed that notifications ‘jeopardised their confidential relationship with their patients, which was enshrined in the ancient Hippocratic oath’ (Mooney, 1999: 240). In addition, physicians argued that notification systems risked the potential for sensitive information to be misused by ‘rivals in the medical marketplace’ or would increase patients’ reluctance to attend a doctor ‘under the fear of exposure’ (Mooney, 1999: 265). Similarly, in New York City, physicians reportedly resented the mandatory notification of tuberculosis in 1897 (and later the notification of venereal disease) which created a tiered system, whereby the names of private (and therefore wealthier) patients were withheld but the names of poorer patients were included with the notification (Bayer and Fairchild, 2000; Fairchild *et al.,* 2003). Despite this early reticence and the limits to a person’s rights to confidentiality and privacy that disease notifications continue to represent, the compulsory collection of notifiable disease data and the application of these data to control disease is widely accepted and considered justifiable in order to protect the public from infectious diseases and ‘contagion’ (Declich and Carter, 1994; Fairchild *et al.,* 2003; Gilbert *et al.,* 2019; Kass, 2001; Lee *et al*., 2012).

In an era when transmission risk behaviours were highly stigmatised or illegal in many countries, the emergence of the HIV/AIDS epidemic raised new concerns about disease notifications and individual rights to privacy (Bayer and Fairchild, 2000). Affected communities and advocates were concerned that notifications containing a person’s name ‘would represent an unjustifiable violation of the right of privacy’ (Bayer and Fairchild, 2000: 12). For example, in the USA, while other diseases (e.g. tuberculosis) required names as part of notifications, for HIV, significant opposition existed due to the stigma and discrimination surrounding HIV positivity and associated risk behaviours (Bayer and Fairchild, 2000; Fairchild *et al.,* 2007; Lee *et al*., 2012). Given its association with homosexuality, many advocates and people with HIV feared that inappropriate access to such information could impact people’s employment prospects, access to housing and other services (Bayer and Fairchild, 2000; Fairchild *et al.,* 2007; Lee *et al*., 2012). Furthermore, the opponents believed ‘that the individual and public health benefits were not sufficient to balance the infringement on individual rights of HIV-infected persons’ (Sweeney *et al.,* 2013: 560–561). In Australia, HIV remains treated differently in the disease notification systems today (a code is entered into government notifications data rather than the person’s full name, with names retained in laboratory testing and with the diagnosing clinician; Department of Health and Aged Care, 2012).

While various international treaties and regulations had existed for much of the 20th century to promote disease notifications and the sharing of public health information, it was not until the 2005 revision of the International Health Regulations (IHR) that rights were connected to disease notifications and surveillance (Fidler, 2005). The new IHR incorporated principles that aligned with international human rights principles (Fidler and Gostin, 2006). For example, the IHR states that these regulations shall be implemented ‘with full respect for the dignity, human rights and fundamental freedoms of persons’ (Plotkin, 2007: 841). The IHR also recognises the importance of confidentiality, that data are only collected for a specific public health purpose, are accurate and kept up to date, kept no longer than necessary and the ‘least invasive and least intrusive’ means are used to protect public health (Plotkin, 2007). It is also worth noting that the IHR is focused on public health risks and events that pose potential harm to the population, rather than actions that pose a benefit. That is, while the IHR provides an excellent framework for consideration of population-level protections, lacking is a consideration of the longer-term health benefits that can be achieved for individuals. For example, for those living with hepatitis C who are unaware that a new effective treatment exists, using notification data to follow them up has the potential to lower their risk of longer-term health impacts.

### Emerging Uses of Notification Data

Disease notifications alert authorities to new cases of a disease or illness, whether known or unknown, and facilitate the monitoring of disease trends and epidemiology. In doing so, these notifications aim to allow authorities to implement strategies to prevent further infections or control the disease (such as through partner notification or contact tracing). However, discussions of alternative uses for these data have evolved—including for the purpose of contacting individuals diagnosed with a disease to guide appropriate care. In some cases, this might include following up those diagnosed with information about treatments that were not available at a time when their notification was made by their diagnosing clinician. For example, in 1985, Thomas Vernon at the Colorado Department of Health argued that ‘reporting would also create the possibility of expeditiously notifying the infected [individual] when effective antiviral therapeutic agents became available’ (Fairchild *et al.,* 2007: 177). With the advent of more effective antiviral agents for HIV—that can be both lifesaving for individuals and achieve a broader public health benefit through the prevention of transmission—Sweeney *et al.* (2013) argue that there has been a shift in the risk versus benefit equation of using HIV disease surveillance data to support patient care. Alongside this, challenges in linking and retaining people in HIV care which consequently ‘restrict the individual and public health benefits of antiretroviral treatment’ are similarly influencing this shift (Sweeney *et al.,* 2013: 562).

With these precedents established in the context of therapeutic advances for HIV, similar considerations are now occurring for hepatitis C. The emergence of highly tolerable, effective and curative DAA therapies in recent years has revolutionised hepatitis C care, reducing people’s risk of developing chronic liver disease, liver failure and liver cancer. It has also advanced ambitions to eliminate hepatitis C as a public health threat. Similar to HIV, the foundation of this ‘elimination’ strategy is based on a treatment-as-prevention strategy. In HIV and hepatitis C, treatment-as-prevention refers to the prevention of transmission to others through treatment when risk behaviours continue (e.g. sharing of injecting equipment). While broader benefits to others at risk of contracting these viruses (e.g. injecting partners) are conveyed through an individual being treated, the person treated also accrues benefits through the prevention of long-term complications of these diseases (Haire and Kaldor, 2013). For hepatitis C, where the outcome of treatment is cure, the individual may also benefit from treatment-as-prevention outcomes through lower risk of reinfection (Sacks-Davis *et al.,* 2024).

Another parallel between HIV and hepatitis C, is that the common route of transmission in countries with concentrated epidemics includes behaviours that are highly stigmatised and, in some cases, illegal such as sex between men and injecting drug use. The use of notification data to help guide diagnosed people into care, therefore makes the issues raised earlier about the balance between privacy and public benefit particularly acute for both HIV and hepatitis C. As with HIV, the use of notification data in this way also introduces additional benefits to individuals with hepatitis C (i.e. through reductions in hepatitis C-related morbidity and death) beyond broader public health and prevention benefits.

An increasing number of projects (both globally and locally in Australia) have been piloting the use of notification systems to increase hepatitis C treatment uptake. For example, in England, a project was launched that aimed to contact over 55,000 people who returned a positive hepatitis C antibody test between 1996 and 2017 for whom there is no record of having been treated (Public Health England, 2018). One of the few studies with published results in the peer-reviewed literature, conducted in New York City, used notification data to contact people who were out of care and had returned a positive antibody result between 2010 and 2015 to encourage further testing (antibody tests only detect lifetime exposure to the hepatitis C virus and a follow-up RNA test is required to diagnose chronic infection) (Webster *et al.,* 2020). The study tested three types of contact intervention (letter only; letter and telephone; and letter, text and telephone) against a no-contact control group. Overall, participants were more likely to receive further testing (e.g. RNA or genotype) in the intervention groups (7.9, 10 and 12 per cent, respectively) compared to the control group (4.6 per cent) (Webster *et al.,* 2020). Amongst the intervention groups, people in the letter and telephone group and letter, text and telephone group had higher odds of receiving further testing than the letter-only group (adjusted odds ratio = 3.57 [95% CI, 1.29–8.85] and adjusted odds ratio = 5.78 [95% CI, 1.98–15.63], respectively) (Webster *et al.,* 2020). However, the authors note that approximately 70 per cent of people could not be contacted in two of the intervention groups (letter and telephone, and letter, text and telephone) and 33 per cent of over 2000 letters were returned to the sender due to incorrect contact details (Webster *et al.,* 2020). In comparison, as part of the Northern Holland Hepatitis Retrieval Project—which aimed to identify all people with a diagnosis of hepatitis B and C recorded over 15 years who had been lost to follow-up care—Dutch privacy regulations prevented direct contact with people who did not have updated contact details (Beekmans and Klemt-Kropp, 2018). As a result, just 24 (5 per cent) of 150 people with hepatitis C who had been lost to follow-up were eligible for further evaluation (Beekmans and Klemt-Kropp, 2018). Some Australian jurisdictions have also been piloting the use of notification data to re-engage people with hepatitis C, however there is limited publicly available evaluation data on the outcomes of these interventions (Walker *et al.,* 2021).

Walker *et al.* (2021) explored the acceptability of using notification data through focus groups with people with lived experience of hepatitis C and/or injecting drug use, one of few published studies to examine this issue. Many focus group participants were unaware that hepatitis C was a notifiable disease and some expressed outrage at the sharing of their personal details with jurisdictional health authorities (Walker *et al.,* 2021). While many participants initially expressed concern about the prospect of receiving contact from a clinician or jurisdictional health authorities, most agreed overall that there were broader benefits associated with using notification data to locate people diagnosed with hepatitis C to inform them about the availability of DAAs and link them into treatment and care (Walker *et al.,* 2021). The authors concluded that there was support for this approach to increase access to hepatitis C treatment, with potential benefits outweighing any potential harms (Walker *et al.,* 2021).

In the context of HIV, Sweeney *et al.* (2013) argue that these uses of disease notification data involve a fundamental paradigm shift away from the initial purposes of compulsory disease notification (disease monitoring and outbreak management) to proactive engagement in care. Despite broadening the potential purpose of disease notification follow-up to include guiding individual care, in addition to informing public health responses, many of the historical tensions associated with compulsory notifications have resurfaced. For example, some physicians have been concerned about potential breaches of patient privacy impacting the physician–patient relationship, the potential for data to be misused and exacerbate stigma and discrimination, and government overreach in the lives of individuals (Buchbinder *et al.,* 2020).

While some argue that these uses of disease notification data are justified and align with the intent of disease surveillance systems (Buchbinder *et al.,* 2020), we must also be cognisant that this is occurring at a time where policy approaches are resulting in a ‘shift *away from* the individual’ with the potential to override consent processes (Seear and Lenton, 2021: 235, emphasis in original). Seear and Lenton (2021) argue that by focusing on broad-scale public health strategies that seek to increase testing, treatment and follow-up there is a risk that some of the needs and interests of individuals (those affected by hepatitis C) may be obscured. Simultaneously, reductions in hepatitis C transmission and prevalence is assumed to be in both the public’s and the individual’s interests (Fraser and Moore, 2011; Seear and Lenton, 2021). However, it can be argued that by reducing the prevalence of hepatitis C in the community, individuals at risk of hepatitis C, such as those who share injecting equipment, derive a direct benefit as a result of ‘treatment-as-prevention’ through the reduction of the risk of contracting hepatitis C and any long-term consequences of it. There is emerging direct evidence of this benefit in the context of sustained and widespread access to DAAs reducing the incidence of primary hepatitis C infection (van Santen *et al.,* 2023) and post-treatment reinfections (Sacks-Davis *et al.,* 2024). Haire and Kaldor (2013) suggest that treatment-as-prevention approaches may be of particular benefit to people who face barriers to adopting preventive behaviours, and may help shift the inequitable burden of disease experienced by certain populations. However, Fraser and Moore (2011: 377) argue that the medicalisation and focus on hepatitis C treatment is ‘individualising and responsibilising’, that is, individuals are ascribed a high degree of responsibility to reduce the risk of disease transmission even though they may be experiencing poverty, marginalisation and inequality and thus may lack the means and resources to do so.

These arguments have similarly been made in the context of HIV, whereby the disease is singled out or is treated as ‘exceptional’ to the extent that it ignores other competing interests of the individual such as employment, housing, alcohol and other drug dependencies (Buchbinder *et al.,* 2020). While some research has found improved physical and mental health, improved social connections and quality of life amongst people who inject drugs who have been treated for hepatitis C (Madden *et al.,* 2018; Perumalswami *et al.,* 2018; Torrens *et al.,* 2020), others have found that hepatitis C treatment is not associated with long-term improvements in patient-reported outcomes (Goutzamanis *et al.,* 2021). Furthermore, as Farrugia *et al.* (2022: 840) illustrate, for a small percentage of people they may not be cured in the first instance resulting in ‘heightened scrutiny from health professionals’ for their perceived treatment ‘failure’. For others who have been cured, hepatitis C may linger in their lives in the form of antibodies, medical records and in their children’s lives, acting as a constant reminder of their past (Farrugia *et al.,* 2022). These findings are important to keep in mind because there is no guarantee that treatment alone will bring immediate change to the lives of all individuals with hepatitis C, despite achieving broader public health gains. In addition, for those with hepatitis C liver cirrhosis, regular and ongoing monitoring is required to reduce their risk of liver cancer (Madden *et al.,* 2018)

In recognition of the problems that the retrospective use of notifications can create, Sweeney *et al.* (2013: 560) call for robust ‘legal and other controls’ that relate to the ‘collection and use (or non-use) of surveillance data’ that balances ‘beneficence, respect for persons, and justice’ (562). However, in the following sections, we seek to highlight limitations associated with rights protections in Australian public health and human rights legislation and raise issues that must be worked through before notification data are used in this way.

## Public Health Legislation

In Australia, a complex legislative framework underpins the mandatory notification of diseases with responsibilities held by the federal government and state and territory governments. The federal government maintains the National Notifiable Disease List under the *National Health Security Act 2007.* One or more cases of any disease on this list, including hepatitis C, is considered to be a ‘public health event of national significance’ under the *National Health Security Act 2007* (Cth) requiring states and territories to collect information and initiate a public health response. State and territory public health legislation requires that all confirmed cases of hepatitis C must be reported (by medical practitioners, pathology services, hospital CEOs and other professionals depending on the jurisdiction) to the respective health department in that state or territory (Walker *et al.,* 2020). This information is then deidentified and shared with the federal government to support national disease surveillance efforts. Therefore, the use of notification data to contact people previously diagnosed with hepatitis C would occur at the state and territory level, given that only deidentified information is shared with the federal government.

### State and Territory Public Health Legislation

State and territory public health legislation tends to be broad in nature and is commonly established with the objectives of protecting and promoting public health. Thus, the role of public health legislation extends across a number of domains, and can include, for example, establishing the role and authority of the Chief Health or Public Health Officer, licensing of premises that conduct invasive procedures (body piercing, tattooing and acupuncture) or disease surveillance and notifications of notifiable diseases (Walker *et al.,* 2020).

The stated objectives of public health legislation in each state and territory are broadly to protect, promote and improve public health, including facilitating ways to detect, manage and control infectious diseases. Not all public health Acts explicitly state the purpose of disease notification or maintaining disease registers. In New South Wales, one of the permitted purposes for establishing a disease register is ‘to facilitate the care, treatment and the follow up of persons who have diseases or have been exposed to diseases’ (*Public Health Act 2010* (NSW) s97:1a). Similarly, in Queensland disease registers are also permitted to ‘take action to prevent or minimise transmission of the notifiable condition’ and so that people ‘may be medically examined and undergo treatment for the notifiable condition’ (*Public Health Act 2005* (QLD) s68). In comparison, in Victoria the purpose of disease registers is to support ‘monitoring, surveillance, investigation or intervention’ of a person with a notifiable disease (*Public Health & Wellbeing Regulations 2019* (VIC) s90b).

While much of this legislation relates to the public’s health, public health laws in most jurisdictions also have guiding principles that relate to notifiable diseases and the rights of individuals that should be considered in implementing the relevant Act. These include the right to privacy (Australian Capital Territory, South Australia, Queensland, Western Australia), confidentiality (South Australia), dignity (South Australia), liberty (Queensland and Western Australia), to be free/protected from discrimination (South Australia, Queensland, Western Australia), the right to receive information about the disease and any treatment (Australian Capital Territory, Victoria and Western Australia), the right to be supported to make an informed decision about medical treatment (South Australia and Queensland) and the right to examination and treatment provided free of charge, if certain criteria are met (Western Australia).^1^^,^^2^ However, these rights are not absolute. For example, in South Australia, the rights of individuals can be overridden by the overriding principle ‘that members of the community have a right to be protected from a person whose infectious state or whose behaviour may present a risk, or an increased risk, of the transmission of a controlled notifiable condition’ (*South Australian Public Health Act 2011* s14). Furthermore, the WA *Public Health Act 2016* makes clear that these rights are not legal rights enforceable in a court of law, and thus there is no remedy for any breach of these rights. Instead, these rights are mainly prescribed to guide decision-making and should be ‘regarded’ in the implementation of the Act.

### Primacy of the Public

There are two key dilemmas created with the application of Australia’s public health legislation to the use of historical notification data. The first is balancing public interests and individual rights; the second involves the inequality and stigma potentially generated and/or exacerbated by actions in response to disease notifications.

First, the ‘primacy of the public’. Regardless of the disease, this legislation sets out that the broader community needs to be protected from the ‘transmitter’ and that the rights of the public or a group override those of an individual. While individual rights (as described in the guiding principles of public health legislation) should be regarded in the implementation of these Acts, these rights are not enforceable and instead serve as principles to guide decision-making. Indeed, various authors have outlined the lack of protections within public health legislation when compared to mental health or guardianship laws despite the significant limitations to a person’s liberty and autonomy that can be exercised under public health legislation (i.e. the power to require a person to submit to testing or treatment, the ability to detain people with a notifiable disease that present an ‘unacceptable risk’ to public health) (Carter, 2020; Stewart *et al.,* 2020; Storrier *et al.,* 2022).

Internationally it has been argued that using notification data to contact people who are not engaged in hepatitis C care supersedes the need for individual informed consent when the objective is in the interests of public health (Andaluz *et al.,* 2020; Buchbinder *et al.,* 2020). The public health benefit is primarily considered to be the reduction of viral transmission and the ability to ‘save lives’ from the long-term consequences of these diseases when left untreated (Buchbinder *et al.,* 2020). Furthermore, Andaluz *et al.* (2020) argue that such approaches are made more justifiable in countries that have strong hepatitis C care programs to which people can be referred. Moreover, the need for informed consent from individuals has been positioned as impractical or unnecessary and outweighed by the broader benefits to individuals and the public (Buchbinder *et al.,* 2020). Indeed, Andaluz *et al.* (2020) argue that acting on hepatitis C notifications when an effective treatment is available could be considered not only ethical but ‘obligatory’.

However, these arguments impose a particular understanding of ‘benefit’ to individuals. While for many people treatment will be beneficial, for others, it may not, and a small percentage may not be able to be cured. This argument also assumes that any benefits to the individual or public outweigh potential costs to individuals, including how affected communities might react to notification data being used for a new purpose, how they might respond to contact from a representative of a health department (or similar) or indeed that notifications data containing personal and sensitive information is collected by jurisdictional health authorities at all (see Walker *et al.,* 2021). Furthermore, these arguments generally assume that a person is unintentionally ‘lost to follow up’ and aligns with Fraser and Moore’s positioning of a person with hepatitis C as a ‘responsible, rational and self-regulating drug-using subject’ (Fraser and Moore, 2011: 376). This is problematic according to Fraser and Moore (2011) as this positioning ignores the broader social context in which such drug use occurs, including stigma and discrimination, the criminalisation of drug use, incarceration, mental illness and homelessness (to name a few). The same argument could also be put to someone living with HIV, based on prevailing community attitudes and social contexts of risk and HIV.

In addition, as Buchbinder *et al.* (2020) note, a person may be intentionally disengaged from services or may not want to be found by authorities for fear of further surveillance. Little space has been given in the available literature for this possibility and how to navigate this situation. Seear and Lenton (2021) therefore caution against waiving the right to informed consent or breaching privacy as this may in fact undermine the hepatitis C elimination agenda altogether through the erosion of trust, and lead to further disengagement from medical care (Seear and Lenton, 2021). Furthermore, privacy protections are fundamental legal protections and should not be waived simply because it may be impractical to seek consent. As Beekmans and Klemt-Kropp (2018) experienced with the Northern Holland Hepatitis Retrieval Project, they were unable to contact people who did not have updated contact details due to Dutch privacy regulations. These regulations are important to offer protection for individuals’ rights to privacy and the use of their personal and sensitive information. Follow-up of notifications should occur within the framework of privacy regulations, which should not be cast aside due to practicalities—whether such practicalities are assumed or otherwise.

It is also problematic to justify that fundamental rights regarding consent and privacy should be waived in countries with hepatitis C care programs, as Andaluz *et al.* (2020) suggest. They do not provide criteria for measuring the apparent strength’ of a country’s hepatitis C program, how compliance with these criteria might be determined and by whom. It seems that Andaluz *et al*.’s (2020) key point may be that the ready availability of hepatitis C care in some countries should bolster the case for extraordinary measures to locate people and offer them treatment. Conceptualising consent, privacy, health information and rights through the narrow lens of health benefits might obscure other vital considerations including ways that health might be put at risk through such practices. In particular, measuring the ‘strength’ of a country’s healthcare system on the basis of resourcing and a perceived conspicuous availability of care may obfuscate underlying weaknesses, including those that manifest in mistrust people have for such systems due to stigma and discrimination they experience in health care. Many people already lack trust in healthcare systems especially digital health systems (Newman *et al.,* 2020). Stigma is a well-known and documented phenomenon among people affected by the virus, even after cure (Kagan *et al.,* 2023). The possibility of exacerbating mistrust and/or stigma must also be taken into account in any discussion about measures designed to encourage people into care. These may signal to weaknesses in a country’s hepatitis C care program and have the potential to undermine the elimination agenda more broadly.

### Inequitable Systems

While Australia’s public health legislation may appear neutral and objective, Kass (2001) reminds us that the disease surveillance systems permitted under this legislation are inherently unfair, because the burden of communicable disease often falls disproportionately on socioeconomically disadvantaged or otherwise marginalised communities. This is true in the case of hepatitis C, where the greatest burden in Australia is borne by people who inject drugs and people currently or previously incarcerated, of which Aboriginal and Torres Strait Islander people are over-represented (Department of Health, 2018; Kirby Institute, 2018). Furthermore, it is well documented that people who inject drugs and people with hepatitis C experience significant stigma and discrimination associated with the disease and/or its connections with injecting drug use (Brener *et al.,* 2016; Heard *et al.,* 2021; Hopwood *et al.,* 2006; Treloar *et al.,* 2013). Such stigma is also commonly perpetuated amongst healthcare workers and within healthcare interactions (Centre for Social Research in Health, 2021; Heard *et al.,* 2021; Hopwood *et al.,* 2006). Thus, any actions that occur in response to these notifications, including those that involve invitations to engage with health systems, risk being felt and distributed unequally and may exacerbate stigma and inequity.

However, at the same time, using notification data to link people to hepatitis C treatment could generate distributive justice for affected communities by reducing an inequitable burden of disease that has largely been driven by the criminalisation of drug use. For example, in 2016, approximately 50 per cent of people entering prison who reported injecting drugs had been exposed to hepatitis C (Butler and Simpson, 2017). Furthermore, state and territory governments have also failed to prevent hepatitis C transmission (including reinfections) in prisons due to a reticence to provide prison-based needle and syringe programs despite these being widely available in the community (Carson *et al.,* 2022).

## Human Rights Legislation

Not acting on notification data may prevent a person benefitting from available treatment and care, thus infringing on their ‘right to life’. While ‘best-practice’ care would dictate that a healthcare worker who ordered a hepatitis C diagnostic test has responsibility for ensuring a patient is informed of the result, we know that this is not always possible and subsequently does not always occur (Burnet Institute and Kirby Institute, 2021). Acting on a notification is particularly pertinent for people who are lost to follow-up (either intentionally or unintentionally) or are unaware that newer treatments are available with fewer side effects and contraindications. Moreover, in the case of hepatitis C, which has a long asymptomatic phase, it may take more than 10 years for cirrhosis (permanent liver damage increasing the risk of liver cancer and liver failure) and symptoms of the disease to develop (Thompson *et al.,* 2013). As a result, it may be considered unethical to not use notification systems for this purpose, given that treatment can prevent serious liver damage and further complications.

While not specifically in the context of hepatitis C, Gilbert *et al.* (2019) argue that mistrust could be generated from the omission of data or not taking action on available data. They argue this ‘would attract public, media and political censure if they failed to prevent or limit an infectious disease emergency because they failed to utilise or respond to intelligence, whatever the source, which could have predicted it’ (Gilbert *et al.,* 2019: 183). Similar arguments have been made about the use of notification data and hepatitis C (Andaluz *et al.,* 2020).

While the non-use of such data poses ethical dilemmas, so too does the *use* of such data, as we have highlighted in the section above. In this section, we draw attention to the limitations of human rights protections in Australia to critically question how decisions are made and justified about the use or non-use of data.

Australia is the only democratic nation without a Charter or Bill of Human Rights (The Human Rights Law Centre, 2019). Some rights are prescribed under the Australian Constitution as well as in legislation at the federal and state/territory levels. The *Human Rights (Parliamentary Scrutiny) Act 2011* operates at the federal level. The human rights covered under this Act include those described in the seven core human rights treaties that Australia has ratified. This Act primarily operates by requiring any bill (proposed new law or amendment to existing law) introduced to Parliament to be scrutinised for its compatibility with human rights treaties. Importantly, even if a bill is found to be incompatible with rights, parliament may still pass the bill into law.

Some states and territories have also introduced Human Rights Acts (Victoria, Queensland and the Australian Capital Territory). These Acts (often referred to as ‘Charters’) recognise a range of rights, including the right to life, the right to privacy and the right to liberty and require these rights to be considered when parliaments make new laws, as well as when public authorities implement or act on them. It is also worth noting that in Victoria and the Australian Capital Territory, these Acts do not include an explicit ‘right to health’. In Queensland, the *Human Rights Act 2019* includes a right to access health services without discrimination and access to emergency medical treatment.

All of these Acts recognise that at times it may be necessary to restrict or limit rights. To do so, the limitation on rights must be reasonable, necessary, justified and proportionate and implemented with the least restrictive means. A statement of compatibility is produced for parliament to debate, that outlines which rights are ‘engaged’, how they may be limited and offering a justification for limiting these rights. As with the federal Act, even if a bill is found to be incompatible with rights, parliaments in Victoria, Queensland and the Australian Capital Territory may still pass the bill into law. As such, these Acts are considered a weak form of human rights protection, both because they allow for rights to be limited, as long as parliaments can produce a justification, and because the justifications offered are often problematic (Seear, 2020; Seear and Mulcahy, 2021). For example, Seear and Mulcahy (2021) found limitations to the rights of people who use alcohol and other drugs are often considered to be justifiable on the grounds that such substances are intrinsically unsafe and that limitations on people’s rights are required in order to protect communities. These justifications homogenise users of alcohol and other drugs and fail to account for the broader social systems and structures in which the use of these substances occurs. Furthermore, Seear and Mulcahy (2021: 16) argue that human rights scrutiny processes ‘reinscribe narrow, problematic and partial accounts of the aetiology and “nature” of social problems’ creating significant limitations in their ability to assess the impact of rights limitations on specific populations.

Thus, we argue that it is not enough to rely solely on existing human rights legislation in Australia to assess the human rights limitations associated with the use or non-use of notification data, as these processes are themselves flawed and are unlikely to function as a robust system for fully assessing and protecting the rights of people with hepatitis C.

## Conclusion

We have sought to explore the tensions and dilemmas that exist when considering the use of disease notification data for the purposes of follow-up and treatment of people with hepatitis C. Our aim was not to conclude whether this approach would be permissible under public health and human rights legislation but to draw attention to the inherent problems that exist within Australia’s legislative framework. We have highlighted that historical tensions between balancing the needs of the public and that of individuals remain today and that there are weaknesses to rights protections for people who have hepatitis C.

In particular, we have argued that it is not sufficient to simply call for legislation and policy controls relating to the use and non-use of notification data. Instead, we must understand how law is made and the values that are prescribed within such legislation. Thus, we argue that before hepatitis C notification data are used to re-engage people with hepatitis C, affected communities must be consulted and involved in all areas of decision-making and in the design of interventions and policy. Affected communities must be able to express their needs and interests (which may or may not align with broader public health goals) and be involved in determining practically how follow-up and contact should be made, and by whom (Walker *et al.,* 2021). Such an approach is critical for producing a solution that is suitable to communities to ensure that their needs and rights are met in a way that does not lose sight of the rights of individuals in the race to eliminate, nor generate new problems and consequences for affected populations.

Finally, it is important to acknowledge that being ‘lost to follow up’ is often a product of wider social and legal forces, including stigma and discrimination (Fraser *et al.,* 2022). While technological public health innovations are warranted, there is also an ongoing need to reduce the stigma and discrimination that people who inject drugs and people with hepatitis C experience to ensure they receive the healthcare they require in a non-stigmatising and non-discriminatory manner.

## Footnotes

1 At the time of writing, notifiable disease legislation in New South Wales, Tasmania and the Northern Territory did not have guiding principles that relate to individual rights.

2 Public Health Act 1997 (ACT) s99, Public Health Act 2005 (QLD) s66, Public Health Act 2016 (WA) s88, Public Health and Wellbeing Act 2008 (VIC) s111, South Australian Public Health Act 2011 (SA) s14.
